# NSUN2 Promotes Head and Neck Squamous Cell Carcinoma Progression by Targeting EMT-Related Gene LAMC2 in an m^5^C-YBX1-Dependent Manner

**DOI:** 10.3390/biomedicines12112533

**Published:** 2024-11-06

**Authors:** Shuojin Huang, Congyuan Cao, Dongxiao Tang, Yiwen Liu, Wanhang Zhou, Lianlian Liu, Xin Zheng, Qianting He, Anxun Wang

**Affiliations:** 1 Department of Oral and Maxillofacial Surgery, The First Affiliated Hospital, Sun Yat-sen University, Guangzhou 510080, China; huangshj5@mail2.sysu.edu.cn (S.H.); caocy@mail2.sysu.edu.cn (C.C.); zhouwh29@mail2.sysu.edu.cn (W.Z.); liullian@mail2.sysu.edu.cn (L.L.); zhengx76@mail2.sysu.edu.cn (X.Z.); heqt3@mail.sysu.edu.cn (Q.H.); 2 Department of Stomatology, The Third Affiliated Hospital, Sun Yat-sen University, Guangzhou 510630, China; tangdx5@mail.sysu.edu.cn; 3 Department of Endodontics, Stomatological Hospital, School of Stomatology, Southern Medical University, Guangzhou 510280, China; liuyw37@mail2.sysu.edu.cn

**Keywords:** NSUN2, mRNA m^5^C modifications, head and neck squamous cell carcinoma, LAMC2, epithelial–mesenchymal transition

## Abstract

Background/Objectives: Head and neck squamous cell carcinoma (HNSCC) is a prevalent and aggressive cancer with high rates of metastasis and poor prognosis. Recent research highlights the role of 5-methylcytosine (m^5^C) in cancer progression. NSUN2, an m^5^C methyltransferase, has been implicated in various cancers, but its role in HNSCC remains elusive. Methods: NSUN2 expression and its impact on HNSCC were analyzed by using clinical samples and bioinformatic analysis. m^5^C-Bis-Seq was used to assess changes in mRNA m^5^C modification and identify downstream targets. Both in vitro and vivo studies were performed to evaluate the impact of NSUN2 manipulation on tumor growth and metastasis. Results: Results indicated that NSUN2 was significantly upregulated in HNSCC tissues compared to normal tissues and was associated with poor prognosis. NSUN2 knockdown led to decreased cell proliferation, migration, and invasion in vitro and reduced tumorigenicity and lymph node metastasis in vivo. m^5^C-Bis-Seq revealed altered m^5^C-modification patterns upon NSUN2 knockdown, with LAMC2 identified as a key downstream target. Conclusions: NSUN2-mediated m^5^C-modification enhanced LAMC2 stability, promoting epithelial–mesenchymal transition (EMT) signaling pathways. These findings demonstrate that NSUN2 promotes the initiation and progression of HNSCC by stabilizing the LAMC2 transcript through m^5^C-dependent mechanisms, offering a promising epitranscriptomic-targeted therapeutic approach for HNSCC.

## 1. Introduction

Head and neck squamous cell carcinoma (HNSCC) is a malignant neoplasm originating from the squamous cells on the mucosal surface in the head and neck region [1]. In 2020, head and neck cancer was estimated to account for about 930,000 new cases and 470,000 deaths globally [2], with HNSCC being the most prevalent histological subtype within head and neck cancer [3]. HNSCC typically manifests a pattern of invasive growth, frequently resulting in regional lymph node or hematogenous metastasis [4]. Despite recent advancements in therapeutic modalities, the etiology and pathogenesis of HNSCC remain unclear, often leading to limited treatment options and unfavorable outcomes, particularly in cases involving local or distant metastasis [5,6].

Numerous studies indicate that epigenetic regulation, including RNA methylation (e.g., N6-methyladenosine, 5-methylcytosine, pseudouridine), is of critical importance in tumorigenesis, progression, and recurrence of a malignant tumor [7,8]. 5-methylcytosine (m^5^C), one of the most prevalent and evolutionarily conserved RNA modifications, was initially identified in abundant and stable tRNA and rRNA [9]. Recent investigations have unveiled that mRNA m^5^C modification can modulate mRNA metabolism and translocation [10,11]. Methylation of mRNA m^5^C plays a critical role in cellular proliferation and differentiation [12,13,14], stress responses [9], and the onset and progression of cancer [10,15] by regulating mRNA stability, translation, or subcellular localization. NOP2/Sun RNA methyltransferase family members 2 (NSUN2) and 6 (NSUN6) are recognized as the most common mRNA methyltransferases (“Writers”) for m^5^C [11,16,17,18]. According to reports, mRNA m^5^C modification sites are categorized into two types: “Type I” m^5^C sites [17,19], which exhibit NSUN2 tRNA substrate characteristics similar to sequences and structures around the tRNA variable loop, and “Type II” m^5^C sites [16,20], which mimic hybrid tRNA-like substrate features recognized by NSUN6. Furthermore, recent studies have discovered that other family members, such as NSUN5 [21] and NSUN7 [22], also possess mRNA m^5^C modification capabilities. m^5^C-modified mRNA can be identified by the reader proteins, including Aly/REF export factor (ALYREF) [11] and Y-box binding protein 1 (YBX1) [23,24], which promote mRNA stability and nuclear translocation.

Through TCGA database analysis and IHC staining of clinical samples, we found that NSUN2, rather than NSUN6, was highly expressed in HNSCC and related to poor prognosis. Therefore, we concentrate on elucidating the oncogenic mechanism of NSUN2 in HNSCC. An increasing body of evidence suggests that NSUN2 participates in the promotion of certain neoplasms, including non-small cell lung cancer [23], bladder cancer [10], prostate cancer [25], esophageal cancer [26], and HNSCC [27,28]. Previous research indicates that NSUN2 expression is upregulated in HNSCC and is related to shorter survival [28]. Its interaction with T-cell activation status influences patient outcomes, indicating its potential as both a prognostic marker and a therapeutic target [27]. However, the potential regulatory mechanisms and clinical significance of NSUN2-mediated mRNA m^5^C modification in HNSCC remain poorly understood.

This research discovered a significant upregulation of NSUN2 in HNSCC tissues compared to paracarcinoma tissues, correlating with a poorer prognosis. Furthermore, we substantiated the inhibitory effects of NSUN2 downregulation on the proliferation, migration, and invasion of HNSCC. Notably, in vivo studies illuminated a marked attenuation in tumorigenicity and lymph node metastatic potential in HNSCC cells following NSUN2 knockdown. Subsequently, employing single-nucleotide resolution analysis, we delineated characteristic alterations in mRNA m^5^C modification in HNSCC cells subsequent to NSUN2 downregulation. Mechanistically, the heightened expression of NSUN2 collaborates with YBX1 (the m^5^C reader protein), thereby augmenting the stability of LAMC2 mRNA in HNSCC. The upregulation of LAMC2 induces carcinogenic epithelial–mesenchymal transition (EMT) pathway signaling. We posit that the NSUN2-m^5^C-LAMC2-EMT signaling axis propels the onset and progression of HNSCC.

## 2. Materials and Methods

### 2.1. Pathological Samples and the Cancer Genome Atlas (TCGA) Database Analysis

A cohort comprising 131 HNSCC samples and 43 paracancerous tissue samples was sourced from the Department of Oral and Maxillofacial Surgery at the First Affiliated Hospital of Sun Yat-sen University (SYSU-TFAH), covering the period from November 2004 to September 2014. Patients undergoing preoperative radiotherapy or chemotherapy were excluded. Pathological diagnoses and clinical–pathological parameters were determined based on the American Joint Committee on Cancer staging criteria (8th edition). Clinical–pathological characteristics of the 131 patients, including gender, age, differentiation, tumor size, lymph node metastasis (LNM), TNM staging, etc., were collected for assessment. All patients underwent follow-up examinations for a minimum of 5 years (until January 2020) or until death, following the initial treatment determination. This study was approved by the Institutional Review Board of SYSU-TFAH (approval num. [2022]229) and adheres to the Declaration of Helsinki.

Analysis of the TCGA database compared the mRNA levels of NSUN2 and LAMC2 in 504 HNSCC samples and 44 paracancerous samples. Survival analysis was conducted for patients with HNSCC with follow-ups exceeding one month based on gene expression data. All these procedures were executed using R software (V 3.6.3) and relevant R packages.

### 2.2. Hematoxylin and Eosin (H&E) and Immunohistochemistry (IHC) Staining

H&E staining was conducted using an H&E staining kit (G1120-100, Solarbio, Beijing, China). For immunohistochemistry, antigen retrieval and blocking were carried out with a 3% H_2_O_2_ solution, and then incubation with the primary anti-NSUN2 (1:200, 20854-1-AP, Proteintech, Rosemont, IL, USA) overnight at 4 °C. Subsequently, the secondary antibody and streptavidin-peroxidase complex kit (SA1022, BOSTER, Wuhan, China) were applied. Subsequently, the sections were stained with 3,3′-diaminobenzidine reagent (BOSTER) and counterstained with hematoxylin. The IHC score was based on the product of the cell score for each staining intensity and the staining intensity score, ranging from 0 to 300 [29].

### 2.3. Cell Culture and Transfection

HOK cells were procured from ScienCell (Carlsbad, CA, USA). CAL27, SCC25, SCC9, SCC15, and HEK293T were obtained from the American Type Culture Collection. HSC3 cells were generously provided by J. Silvio Gutkind (Bethesda, MD, USA). All cells were incubated at 37 °C in a humidified atmosphere with 5% CO_2_.

Plasmids GV493-shNSUN2, GV493-shLAMC2, and GV505-LAMC2 were produced by GeneChem Co. (Shanghai, China). Plasmids pLVX-NSUN2-WT and pLVX-NSUN2-MUT were produced by GeneCFPS Co. (Wuxi, Jiangsu, China). The lentiviral vectors, along with packaging vectors psPAX2 and pMD2.G, were co-transfected into HEK293T for lentivirus production. The collected virus was used to infect HSC3 and SCC25 cells. After 14 days of incubation in media containing 3 μg/mL puromycin (Beyotime, Shanghai, China), puromycin-resistant colonies were selected, and knockdown efficiency was confirmed by Western blot and qRT-PCR. YBX1 was knocked down using siRNA (Ribobio, Guangzhou, China). The shRNA and siRNA sequences are listed in Supplementary Table S1.

### 2.4. Western Blot Analysis

Western blotting was performed according to our previous protocol [30]. The membranes were incubated with primary antibodies including NSUN2 (1:10,000, 20854-1-AP, Proteintech, Rosemont, IL, USA), LAMC2 (1:500, ab210959, Abcam, Cambridge, UK), YBX1 (1:1000, ab76149, Abcam), Vimentin (1:5000, 10366-1-AP, Proteintech), N-cadherin (1:8000, 22018-1-AP, Proteintech), E-cadherin (1:5000, 20874-1-AP, Proteintech), and GAPDH (1:20,000, 10494-1-AP, Proteintech). Subsequently, the membranes were incubated with anti-mouse secondary antibody (1:5000, SA00001-1, Proteintech) or anti-rabbit secondary antibody (1:5000, SA00001-2, Proteintech).

### 2.5. m^5^C-Bis-Seq, mRNA-Seq, and Bioinformatics Analysis

m^5^C-Bis-Seq and mRNA-Seq were conducted by Cloudseq Biotech Inc. (Shanghai, China) following the manufacturer’s guidelines. m^5^C-Bis-Seq and bioinformatics analysis were conducted as previously described [11]. In brief, rRNA-depleted RNA underwent bisulfite conversion and purification. RNA libraries were then constructed and sequenced on an Illumina Novaseq instrument with paired-end reads of 150 bp. Following 3′-adapter trimming, Cutadapt software (v1.9.3) was used to remove low-quality reads. Next, the meRanGs software (one component of meRanTK, v1.3.0) was applied to align the clean reads to the UCSC hg19 genome, and the meRanCall software (one component of meRanTK) was used to identify m^5^C sites within the genome. Differential methylation sites (DMS) were identified with meRanCompare (one component of meRanTK). Sites with a coverage depth (methylated C value + unmethylated C value) ≥10, m^5^C methylation level ≥ 0.1, and methylated cytosine depth ≥ 2 were considered reliable. Finally, the metaPlotR software (https://github.com/olarerin/metaPlotR) was used to visualize the distribution of m^5^C sites.

For mRNA-Seq, three independent biological replicates were prepared for each experimental condition (shNSUN2#1). Raw counts were obtained using HTSeq software (v0.9.1), and normalization was performed with edgeR. Differentially expressed mRNAs were identified based on an adjusted *p*-value (<0.05) and log2 fold change (≥1). m^5^C-Bis-Seq and mRNA-Seq data are available in the Genome Sequence Archive (GSA) database under accession number HRA007987.

### 2.6. Cell Proliferation, Colony Formation, Wound Healing, Migration, and Invasion Assay

Cell proliferation, colony formation, and wound healing assay were employed according to our previous protocol [30]. Cell proliferation was assessed at predetermined time points using the Cell Counting Kit-8 (CCK-8) (Dojindo, Tokyo, Japan). For the migration assay, 200 μL of cell suspension was added at a density of 5 × 10^4^ cells per well to the upper chamber of Transwell inserts (Corning, Shanghai, China) with DMEM/F12 without FBS. In the lower chamber, 500 μL of DMEM/F12 was added with 1% FBS. For the invasion assay, the chambers were pre-coated with 5% Matrigel (BioCoat, Beijing, China). After adding cells as described for the migration assay, the migrated or invaded cells were fixed after 48 or 72 h, stained with crystal violet, and quantified in five random fields under a microscope.

### 2.7. 5-Ethynyl-2′-Deoxyuridine (EdU) Assay

The effect of NSUN2 on HNSCC cell proliferation was assessed using the EdU detection kit (RiboBio, Guangzhou, China). A total of 10,000 cells were seeded per well in a 96-well plate in triplicate. EdU (50 μM) was added and cells were incubated for 2 h at 37 °C after transfection. Next, cells were fixed with 4% paraformaldehyde for 30 min, permeabilized with 0.1% Triton X-100 for 10 min, and stained with EdU Apollo643 for 30 min and Hoechst 33342 for 20 min. A fluorescence microscope was used to observe and quantify the proportion of EdU-positive cells.

### 2.8. RNA Extraction and Quantitative Real-Time PCR (qRT-PCR)

qRT-PCR was performed according to our previous protocol [30]. Primer sequences used in this study are provided in Supplementary Table S1.

### 2.9. Dot Blot Assay

Total RNA was denatured at 90 °C for 3 min, spotted onto a nylon membrane (Beyotime, Shanghai, China), and cross-linked using ultraviolet light (125 mJ/cm^2^). Following blocking with 5% non-fat milk, the membrane was incubated overnight at 4 °C with anti-m^5^C antibody (1:5000, 68301-1-lg, Proteintech), followed by incubation with an enzyme-linked secondary antibody. Subsequently, the membrane was stained with methylene blue (0.02%) to verify uniformity of RNA levels.

### 2.10. RNA Stability Assay

RNA stability in NSUN2-stably knocked down cells was measured by treating SCC25/HSC3 cells with actinomycin D (5 μg/mL). Subsequently, total RNA was collected at specified time points (0, 2, 4, and 8 h) for qRT-PCR. The half-life (t1/2) was calculated as the time required for a 50% reduction in mRNA expression.

### 2.11. Xenograft Tumor Models

Male BALB/c nude mice (5–6 weeks old, n = 48) were randomly assigned to eight groups (simple randomization method): shCtrl, shNSUN2, NSUN2-OE, NSUN2-MUT, Vector, shCtrl+Vector, shNSUN2+Vector, and shNSUN2+LAMC2, with 6 biological replicates per group in accordance with the 3R principles (Replacement, Reduction, and Refinement). For subcutaneous xenograft models, approximately 1.2 × 10^7^ HSC3 cells diluted in 150 μL PBS were injected subcutaneously into the right axilla under 2% pentobarbital sodium anesthesia to establish subcutaneous xenograft models derived from the cell line. For tongue orthotopic xenograft models, under anesthesia, approximately 1 × 10^6^ cells in 50 μL PBS were injected into the left tongue to establish heterotopic transplantation models. Mouse body weight was measured every 3 days after 1 week. Finally, mice were euthanized by cervical dislocation under 2% pentobarbital sodium anesthesia to harvest subcutaneous xenografts or tongues. Tissues were embedded, sliced, and stained. All animal experiments were conducted at the Experimental Animal Center of SYSU-TFAH, and all animal care and experimental protocols were approved by the Institutional Animal Care and Use Committee of the Clinical Research and Animal Experimentation Ethics Committee of SYSU-TFAH (approval num. [2023]176). When mice met the humane endpoint criteria based on tumor size and overall health status, they were euthanized. Primary outcomes of mouse body weight and tumor volume were independently measured by two researchers (S.H. and D.T.), and the average of the measurements was taken to reduce the influence of confounding factors.

### 2.12. Statistical Analysis

Unless otherwise specified, results were presented as the mean ± SD of biological replicates. The significance of differences in unpaired parameters between two groups was assessed using two-tailed Student’s *t*-tests or chi-square tests for tumor grades. Survival analysis was performed using Kaplan–Meier curves and log-rank tests. The correlation between NSUN2 expression levels and IHC scores was analyzed using Pearson correlation tests. A threshold of *p* < 0.05 was defined as the level of statistical significance. Data management and statistical analyses were conducted using GraphPad Prism 9.5 software (GraphPad Software, San Diego, CA, USA) and SPSS 27 software (IBM SPSS, Armonk, NY, USA).

## 3. Results

### 3.1. NSUN2 Is Upregulated in HNSCC and Related to Poor Prognosis

**Figure 1:**
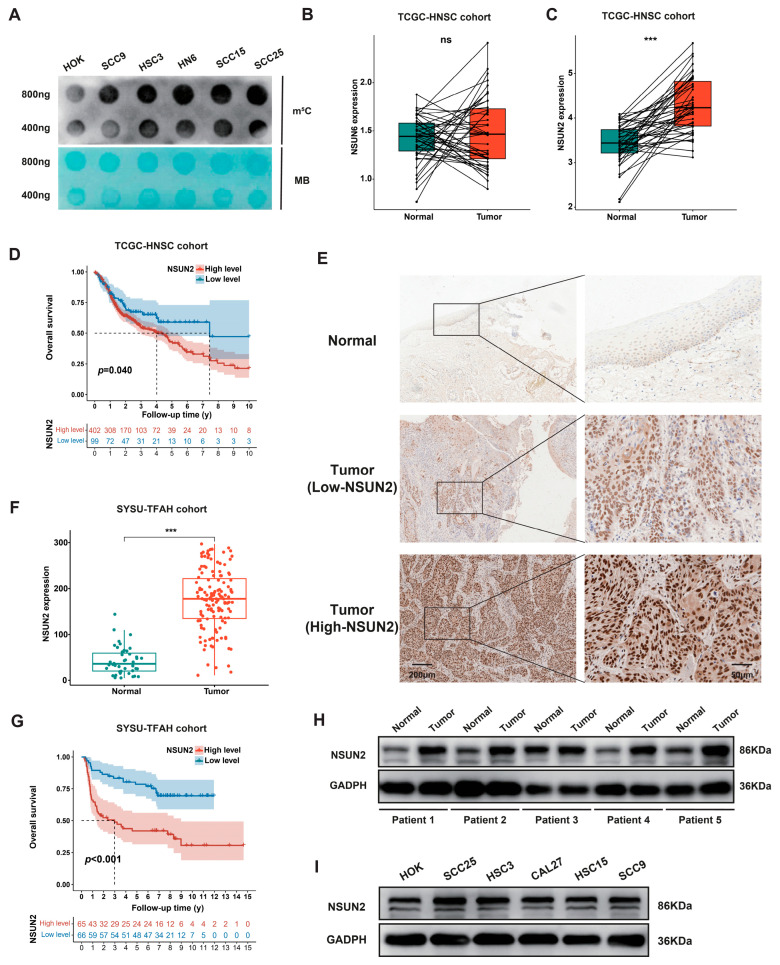
NSUN2 is upregulated and associated with poor prognosis in patients with HNSCC. (**A**) Dot blot analysis displaying the RNA m^5^C hypermethylation tendency in HOK and five HNSCC cell lines. (**B**,**C**) The expression levels of NSUN6 and NSUN2 in the TCGA-HNSC cohort (43 pairs). (**D**) K–M curves of the OS of 501 patients stratified by NSUN2 expression level from the TCGA-HNSC cohort. (**E**) Representative images of NSUN2 expression in normal and tumor tissues in HNSCC examined by IHC. Data were obtained from the SYSU-TFAH cohort. (**F**) The expression levels of NSUN2 in the SYSU-TFAH cohort (131 Tumor, 43 Normal). (**G**) K–M curves of the OS of 131 patients from the SYSU-TFAH cohort. (**H**,**I**) Western blot analysis displaying NSUN2 overexpression tendency in (**H**) five patients, (**I**) HOK and five HNSCC cell lines. ns means not considered statistically significant, *** means *p* < 0.001.

To investigate the potential mechanism of m^5^C mRNA methylation modification in the progression of HNSCC, we conducted dot blot analysis using anti-m^5^C antibodies. The results indicated a significant upregulation of m^5^C levels of HNSCC cells compared to HOK cells (Figure 1A). Employing the TCGA dataset, the mRNA expression levels of two primary mRNA m^5^C methyltransferases, NSUN2 and NSUN6, in 43 pairs of adjacent and cancer tissues from patients with HNSCC was investigated. Notably, only NSUN2 exhibited a markedly higher expression in HNSCC tissues compared to normal tissues (Figure 1B,C). Further analysis indicated that patients with high NSUN2 had a significantly poorer prognosis than those with low NSUN2 (Figure 1D). Subsequent evaluation of NSUN2 protein levels in 174 clinical samples (131 tumors and 43 paracancerous tissues) from our hospital (SYSU-TFAH cohort) using IHC staining demonstrated predominant expression in the cancer cell nucleus and partial expression in the cytoplasm within HNSCC tissues (Figure 1E). Compared to control adjacent tissues, NSUN2 was significantly upregulated in tumor tissues (Figure 1E,F). In the SYSU-TFAH cohort, 131 tumor samples were categorized into low-NSUN2 and high-NSUN2 groups based on immunohistochemistry scores (Table 1). Kaplan–Meier curves indicated a markedly reduced overall survival period in the high-NSUN2 group compared to the low-NSUN2 group (Figure 1G), which is consistent with TCGA database analysis. Western blot results similarly demonstrated a significant elevation of NSUN2 expression levels in tumor samples compared to normal samples (Figure 1H), and in HNSCC cells compared to HOK cells (Figure 1I). Overall, our data suggest that NSUN2 is highly expressed in HNSCC and may be of critical importance in oncogenic regulation.

### 3.2. NSUN2 Affects the Proliferation and Metastasis of HNSCC In Vitro and In Vivo

**Figure 2:**
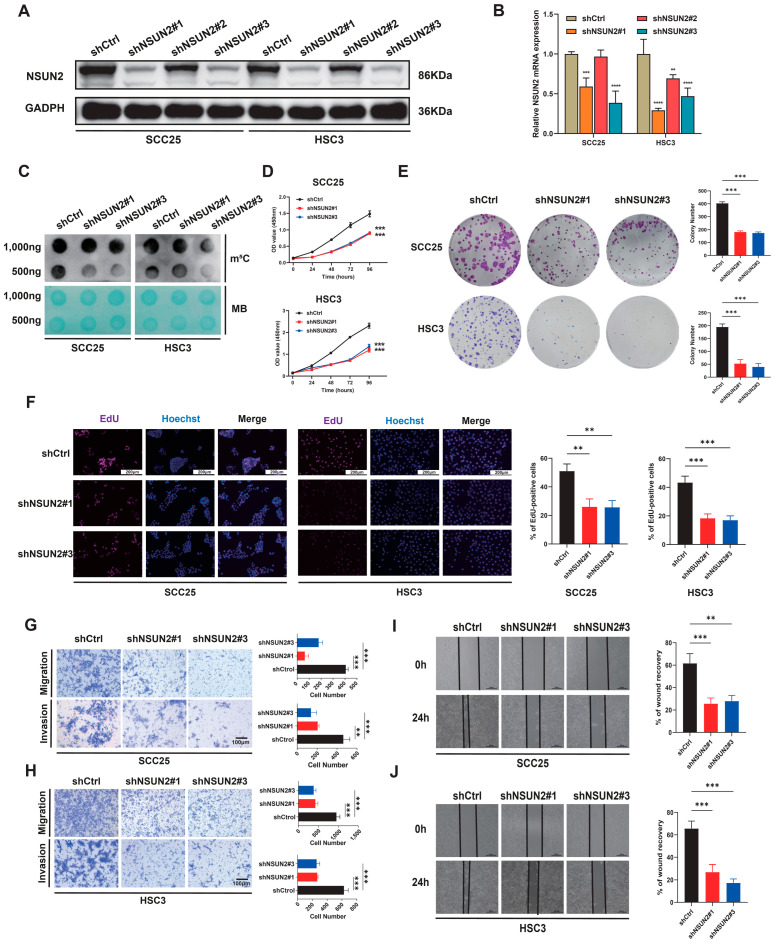
Knockdown of NSUN2 inhibits HNSCC cell proliferation, migration, and invasion in vitro. Western blot (**A**) and RT-qPCR (**B**) confirmed the knockdown of NSUN2 by lentiviral constructs in HNSCC cell lines. (**C**) Dot blot analysis displaying the RNA m^5^C hypermethylation tendency in cells with stable NSUN2 knockdown. (**D**) Cell viability was determined by CCK-8 in cells with stable NSUN2 knockdown. (**E**) Colony formation assays. (**F**) EdU staining assays were performed to evaluate cell proliferation. (**G**,**H**) Migration ability and invasion ability of HNSCC cells with NSUN2 silence were evaluated by Transwell assays. (**I**,**J**) Wound-healing assay. MB means methylene blue, ** means *p* < 0.01, *** means *p* < 0.001, **** means *p* < 0.0001.

To clarify the biological role of NSUN2 in HNSCC, we established NSUN2-silenced SCC25 and HSC3 cell lines via lentiviral transduction, and the knockdown efficiency was validated through Western blot analysis and RT-qPCR. As shown in Figure 2A,B, shNSUN2#2 exhibited low knockdown efficiency, possibly due to off-target effects, poor shRNA design, or inefficient delivery, and was therefore excluded from further experiments. shNSUN2#1 and shNSUN2#3 were selected for subsequent experiments. Dot blot experiments revealed a decrease in overall m^5^C modification levels in RNA from HNSCC cells upon NSUN2 knockdown (Figure 2C). Functional assays (CCK-8, EdU, and colony formation assays) demonstrated a significant attenuation of proliferation and clonogenic capacity in HNSCC cells following NSUN2 downregulation (Figure 2D–F). Furthermore, as adverse prognosis is often attributed to tumor metastasis, we conducted Transwell and wound healing experiments and revealed that NSUN2 knockdown significantly inhibited the migration and invasion activity of SCC25 and HSC3 cells (Figure 2G–J).

**Figure 3:**
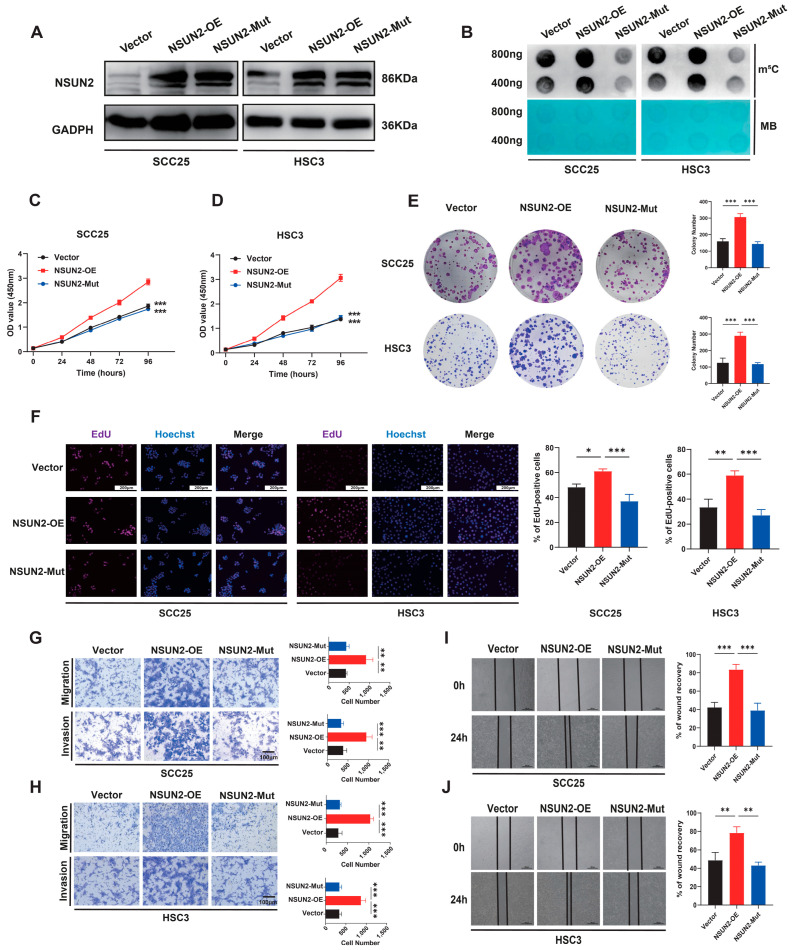
Overexpression of NSUN2 promotes HNSCC cell proliferation, migration, and invasion in vitro. (**A**) Western blot confirmed the overexpression of NSUN2 by lentiviral constructs in HNSCC cell lines. (**B**) Dot blot analysis displaying the RNA m^5^C hypermethylation tendency in cells with stable NSUN2 overexpression. (**C**,**D**) Cell viability was determined by CCK-8 in cells with stable NSUN2 overexpression. (**E**) Colony formation assays. (**F**) EdU staining assays were performed to evaluate cell proliferation. (**G**,**H**) Migration ability and invasion ability of HNSCC cells with NSUN2 overexpression were evaluated by Transwell assays. (**I**,**J**) Wound-healing assay. MB means methylene blue, * means *p* < 0.05, ** means *p* < 0.01, *** means *p* < 0.001.

Subsequently, plasmids expressing wild-type NSUN2 (NSUN2-OE) and its catalytically inactive mutant (NSUN2-Mut, C271A and C321A) were constructed and transfected into HNSCC cells (Figure 3A). Dot blot experiments revealed an increase in overall m^5^C modification levels in RNA upon overexpression of the NSUN2-OE group, but not the NSUN2-Mut group (Figure 3B). Additionally, functional assays indicated that the NSUN2-OE group promoted proliferation and clonogenic capacity in HNSCC cells, while the NSUN2-Mut group had minimal impact (Figure 3C–F). Meanwhile, NSUN2 overexpression significantly enhanced the invasion and migration activity of HNSCC cells, with the catalytically inactive NSUN2 mutant having little effect (Figure 3G–J).

**Figure 4:**
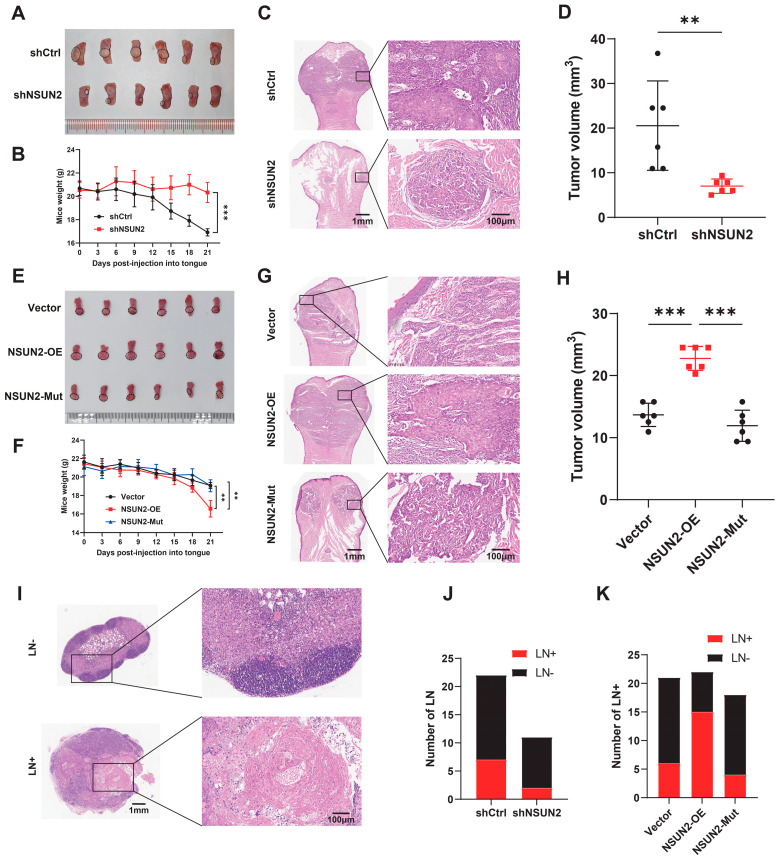
NSUN2 induces proliferation and facilitates metastasis in vivo. (**A**,**E**) Knockdown and overexpression of NSUN2 dramatically influenced HSC3 tumor cell growth in nude mice’s tongue (n = 6). (**B**,**F**) The change in mouse weight after injection into tongue (n = 6). (**C**,**G**) H&E analysis of the tongue tissue of each group. (**D**,**H**) Tumors extracted after 21 days were weighed (n = 6). (**I**–**K**) H&E staining and analysis of cervical lymph node metastasis. ** means *p* < 0.01, *** means *p* < 0.001.

We further utilized a tongue orthotopic transplantation nude mouse model to study the importance of NSUN2 in HNSCC progression. The results indicated that NSUN2 knockdown significantly suppressed tumorigenicity in mice transplanted with HSC3 cells (Figure 4A–D), and H&E staining confirmed reduced neck lymph node metastasis after NSUN2 knockdown (Figure 4J). Conversely, NSUN2 overexpression significantly promoted tumorigenicity in mice transplanted with HSC3 cells (Figure 4E–H) and enhanced lymph node metastasis capability (Figure 4K), while the catalytically inactive NSUN2 mutant had minimal impact.

In summary, our results indicate that the malignant phenotype-promoting function of NSUN2 in HNSCC is dependent on its methyltransferase activity, strongly supporting the role of NSUN2-mediated mRNA m^5^C modification in regulating the progression of HNSCC.

### 3.3. Characterizing the mRNA m^5^C Profile of HNSCC Cells Using mRNA-Seq and m^5^C-Bis-Seq Technology

**Figure 5:**
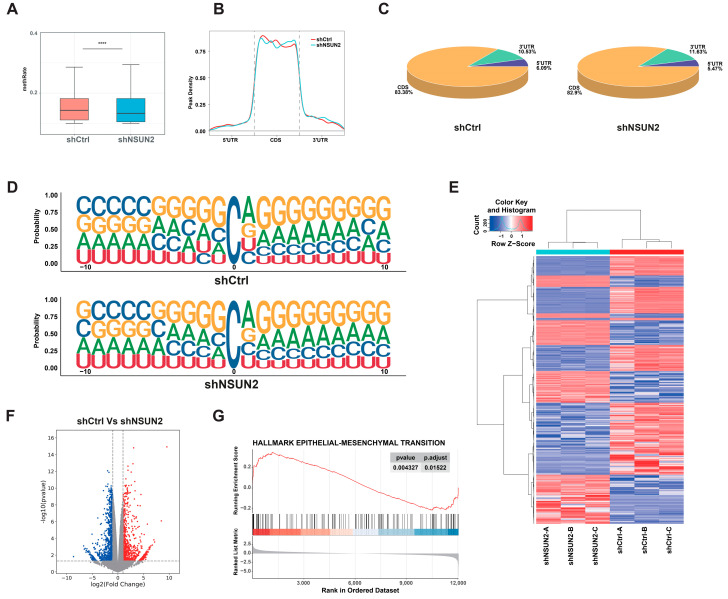
Characterization of mRNA m^5^C-bis-seq and mRNA-seq in HNSCC cells. (**A**) The average m^5^C level was analyzed via m^5^C-Bis-Seq in cells with or without NSUN2 knockdown. (**B**,**C**) The m^5^C distributions within different regions in control or NSUN2 knockdown SCC25 cells. (**D**) The sequences are proximal to the mRNA m^5^C sites in cells with or without NSUN2 knockdown. (**E**,**F**) Heatmap and volcano plot of the differentially expressed genes (DEGs) between cells with and without NSUN2 knockdown. (**G**) GSEA analysis of EMT pathway. **** means *p* < 0.0001.

Given that NSUN2 is one of the m^5^C mRNA methyltransferases, we investigated whether NSUN2 affects mRNA m^5^C modification levels in HNSCC cells. Through m^5^C-Bis-Seq, we identified 11,926 m^5^C sites among 5410 transcripts. Compared to shCtrl, m^5^C methylation levels were significantly decreased in SCC25 cells transfected with shNSUN2 (Figure 5A), indicating the methyltransferase activity of NSUN2 in mRNA m^5^C modification in HNSCC cells. Among these 11,926 m^5^C sites, 83 sites in 80 transcripts were hypermethylated, and 113 sites in 106 transcripts were hypomethylated in the shNSUN2 group compared to the control group. In HNSCC cells, m^5^C sites were distributed across all regions of mRNA, with similar distribution patterns in both groups, and the m^5^C methylation pattern was predominantly located in the coding sequence (CDS) of mRNA transcripts (Figure 5B,C). Interestingly, sequence frequency analysis indicated that most mRNA m^5^C sites were mainly enriched in the CG context and a higher frequency of CAGGGG was observed downstream of m^5^C sites, which is consistent with previously reported findings [11] (Figure 5D). For mRNA-seq analysis, the heatmap and volcano plot of differentially expressed genes (DEGs) in SCC25 cells after NSUN2 knockdown are shown in Figure 5E,F. GSEA analysis proved significant enrichment of downregulated genes in the EMT pathway (Figure 5G).

### 3.4. Identification of LAMC2 as a Downstream Target of NSUN2 in HNSCC

**Figure 6:**
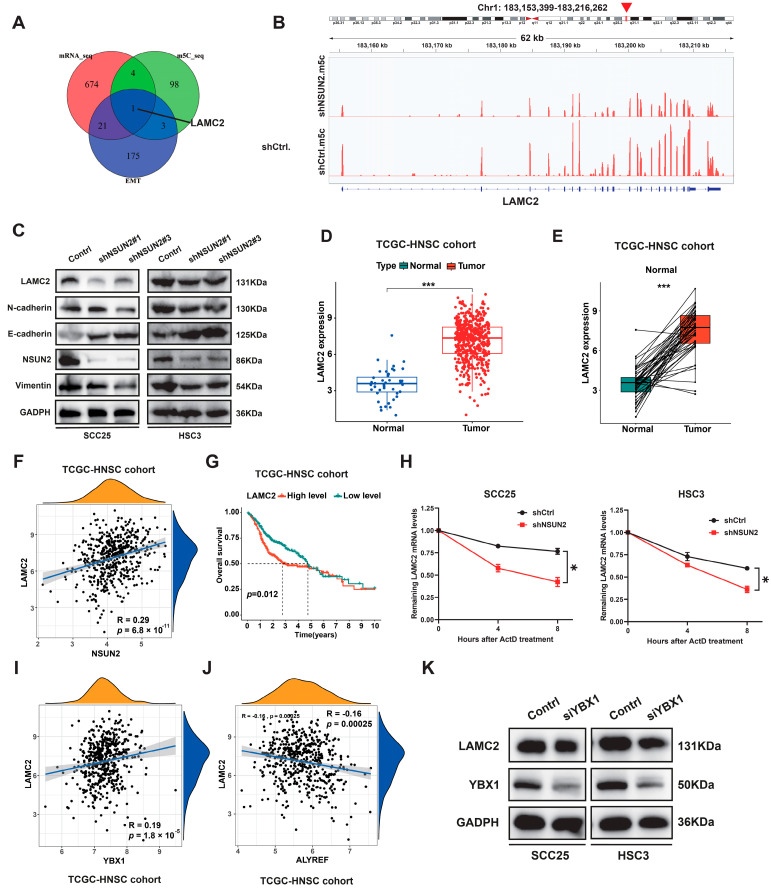
LAMC2 was identified as a downstream target of NSUN2. (**A**) Venn diagram showing the selection for the downstream target of NSUN2 according to RNA-seq, m^5^C-seq, and EMT pathway genes. (**B**) The LAMC2 mRNA m^5^C sites in cells with or without NSUN2 knockdown. (**C**) NSUN2 silence could suppress LAMC2 and EMT-related gene expression. (**D**,**E**) The expression levels of LAMC2 in the TCGA-HNSC cohort (504 Tumor, 44 Normal, 43 pairs). (**F**) A positive correlation between LAMC2 and NSUN2 mRNA expression in HNSCC based on TCGA data. (**G**) K–M curves of the OS of 504 patients stratified by LAMC2 expression level from the TCGA-HNSC cohort. (**H**) The relative LAMC2 mRNA expression upon NSUN2 knockdown in SCC25 and HSC3 cells treated with 5 μg/mL actinomycin D for indicated times. (**I**) A positive correlation between LAMC2 and YBX1 mRNA expression in HNSCC based on TCGA data. (**J**) The correlation between LAMC2 and ALYREF mRNA expression in HNSCC based on TCGA data. (**K**) LAMC2 protein expression in cells transfected with YBX1 siRNA. * means *p* < 0.05, *** means *p* < 0.001.

We analyzed m^5^C-Bis-Seq and mRNA-seq data in HNSCC cells, focusing on genes related to the EMT pathway, to investigate potential targets of NSUN2. Interestingly, LAMC2 was identified as a potential downstream target of NSUN2 (Figure 6A) with associated m^5^C modification sites (Figure 6B). Western blotting confirmed decreased expression of LAMC2 and EMT-related molecules (N-cadherin, Vimentin) following NSUN2 knockdown (Figure 6C). TCGA database analysis revealed elevated expression of LAMC2 in HNSCC (Figure 6D,E), positively correlating with NSUN2 expression (Figure 6F). Kaplan–Meier analysis indicated that the overall survival of the high-LAMC2 group was significantly worse than that of the low-LAMC2 group (Figure 6G).

We further investigated whether NSUN2 regulates the expression of LAMC2 through affecting the stability of its mRNA. After actinomycin D treatment, LAMC2 transcripts in NSUN2-knockdown cells exhibited a shorter half-life (Figure 6H), indicating a significant reduction in LAMC2 mRNA stability. The stability of m^5^C-modified mRNA is predominantly regulated by YBX1 [23] and ALYREF (m^5^C readers) [31,32]. Consequently, we explored whether YBX1 or ALYREF affects the expression of LAMC2 in HNSCC. Analysis of the TCGA database revealed that, in HNSCC, YBX1 (Figure 6I), rather than ALYREF (Figure 6J), is positively correlated with LAMC2 mRNA expression. Using siRNA targeting YBX1, we observed a reduction in expression of LAMC2 (Figure 6K).

### 3.5. NSUN2-m^5^C-LAMC2-EMT Axis Mediates Proliferation, Migration, and Invasion of HNSCC Cells In Vitro and In Vivo

**Figure 7:**
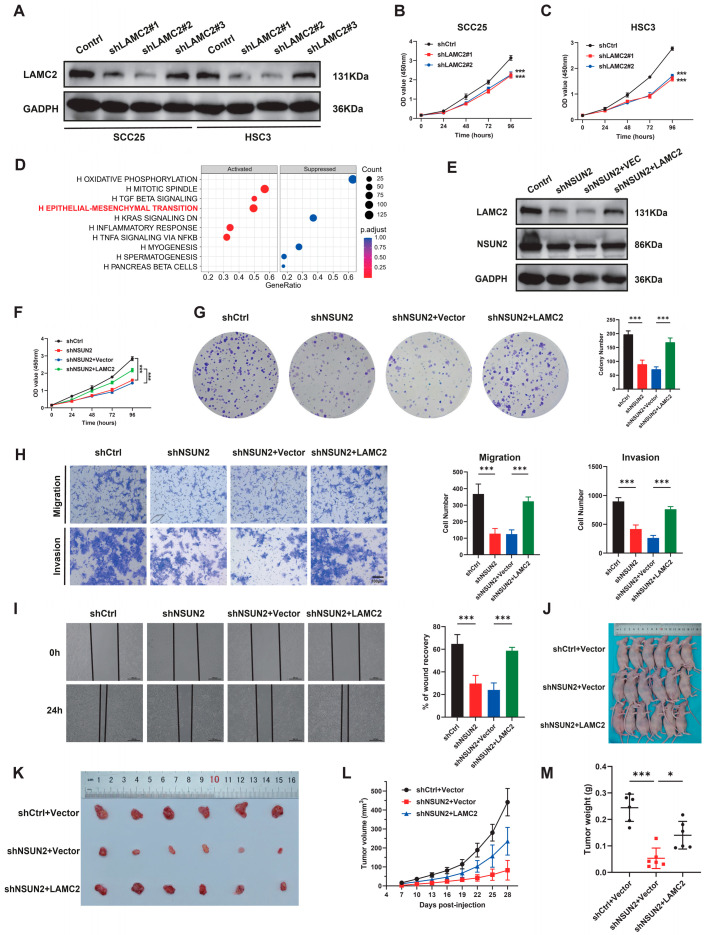
LAMC2 mediates NSUN2-induced cell proliferation, migration, and invasion of HNSCC cells in vitro and in vivo. (**A**) Western blot confirmed the knockdown of LAMC2 by lentiviral constructs in HNSCC cell lines. (**B**,**C**) Cell viability was determined by CCK-8 in cells with stable LAMC2 knockdown. (**D**) GSEA analysis of differentially expressed genes (DEGs) in the high-LAMC2 group of TCGA database. (**E**) Western blot presenting the effect of LAMC2 overexpression in NSUN2 knockdown HNSCC cells. CCK-8 (**F**), Colony formation assays (**G**), Transwell assays (**H**), and Wound-healing assay (**I**). (**J**,**K**) Tumors extracted from nude mice with different treatments are shown (n = 6). (**L**) Tumor volume was measured every 3 days (n = 6). (**M**) Tumors were weighed after mice were sacrificed (n = 6). ns means not considered statistically significant, * means *p* < 0.05, *** means *p* < 0.001.

To further investigate the impact of LAMC2 on NSUN2-induced cancer progression, we established SCC25 and HSC3 cell lines with LAMC2 knockdown. Western blot analysis was used to confirm transfection efficiency (Figure 7A). CCK-8 assays demonstrated that the downregulation of LAMC2 significantly decelerated the proliferation of HNSCC cell lines (Figure 7B,C). Analysis of the TCGA database revealed that DEGs in the high-LAMC2 group of patients with HNSCC were significantly enriched in the EMT pathway (Figure 7D).

Subsequently, we induced overexpression of LAMC2 in NSUN2-depleted HNSCC cells (Figure 7E). Results revealed partial rescue of proliferation, migration, and invasion capabilities in NSUN2-depleted HNSCC cells through LAMC2 overexpression (Figure 7F–I). Following this, the impact of ectopic expression of LAMC2 in NSUN2-depleted HNSCC cells was assessed by using a xenograft tumor model. Results indicated that the cancer growth in the shNSUN2+Vector group was significantly slower than in the shCtrl+Vector group, while the shNSUN2+LAMC2 group partially rescued the growth of HNSCC cells in vivo (Figure 7J–M). In summary, these findings suggest that NSUN2-mediated RNA 5-methylcytosine promotes the progression of HNSCC by enhancing the stability and expression of LAMC2 mRNA.

## 4. Discussion

In recent years, numerous RNA modifications have been elucidated [33]. As a pivotal epigenetic modification of RNA, m^5^C is implicated in various regulatory mechanisms and biological functions, encompassing cancer [25,26], neurodevelopmental disorders [34,35], inflammation [36], and infertility [37]. This study is the first to use m^5^C-Bis-Seq to comprehensively analyze the distribution characteristics of m^5^C modifications in HNSCC, revealing elevated m^5^C modifications and increased NSUN2 expression levels. Furthermore, m^5^C-Bis-Seq and mRNA-Seq analyses identified LAMC2 as a potential target regulated by NSUN2 in promoting EMT in HNSCC, providing novel insights into how NSUN2 modulates the m^5^C landscape and facilitates cancer progression through the stabilization of LAMC2 mRNA.

The mRNA m^5^C modification is catalyzed by two methyltransferases, NSUN2 and NSUN6 [11,16]. Notably, NSUN2 has garnered increased attention due to its carcinogenic role in various cancer types [10,23,25,26,27,38,39]. The study by Lu L. et al. [27] reported that the interaction between NSUN2 expression and T-cell activation status influences the survival of patients with HNSCC, but did not explore NSUN2’s role in m^5^C modification within HNSCC. Additionally, other studies have suggested a potential role for NSUN2 in m^5^C modification in HPSCC [38] and NPC [39], though none utilized m^5^C-seq to analyze its impact on the m^5^C modification landscape in HNSCC cells. Building on previous research, this study is the first to emphasize the m^5^C-dependent oncogenic role of NSUN2 in HNSCC through m^5^C-Bis-Seq. NSUN2 is aberrantly overexpressed in HNSCC and significantly correlates with poor prognosis. In vitro, NSUN2 promotes the malignant phenotype of HNSCC cells through its methyltransferase activity. In vivo data show that silencing NSUN2 markedly suppresses HNSCC tumorigenesis and metastasis.

Moreover, to explore the oncogenic mechanism of NSUN2-mediated m^5^C modification in HNSCC, we present the mRNA m^5^C landscape in HNSCC. Remarkably, genes with highly methylated m^5^C in HNSCC tumors are significantly enriched in the EMT pathway. The abnormal activation of EMT signaling pathway is well documented in the development and progression of tumors [40]. In this study, the abnormal expression of NSUN2 was demonstrated to trigger the abnormal activation of EMT signaling through stabilizing LAMC2 mRNA. LAMC2, a subunit of the heterotrimeric glycoprotein laminin-332, is a fundamental component of the epithelial basement membrane, regulating cell movement and adhesion [41]. Intriguingly, we found that the abnormal m^5^C modification in LAMC2 is linked to occurrence and progression in HNSCC through the activation of the EMT pathway. NSUN2 silencing attenuates EMT signal transduction by reducing m^5^C modification levels in LAMC2 mRNA, while LAMC2 overexpression reverses the inhibitory effect on the malignant cell phenotype in NSUN2-depleted cells, highlighting LAMC2 as a key mediator induced by NSUN2 in HNSCC. These findings propose the NSUN2-m^5^C-LAMC2-EMT axis as pivotal in the development of HNSCC, offering a novel regulatory model for NSUN2-mediated HNSCC progression.

Additionally, numerous reports indicate a correlation between elevated LAMC2 expression and adverse prognosis in cancer [42,43,44]. Our results align with these studies, showing upregulation of both mRNA and protein levels of LAMC2 in HNSCC tumors, predicting unfavorable outcomes. This study expands the regulatory mechanisms of LAMC2 expression from an epigenetic perspective, revealing a new mechanism of m^5^C modification regulation in LAMC2 expression, suggesting that downregulating m^5^C modification in LAMC2 may have therapeutic implications for HNSCC. Finally, several studies report that YBX1 is an m^5^C-methylated RNA recognition protein, stabilizing mRNA [23,45]. Surprisingly, upon YBX1 depletion, there is a noticeable reduction in the expression levels of LAMC2, indicating that YBX1 may bind and stabilize m^5^C-modified LAMC2 mRNA.

This research provides significant insights into the role of NSUN2-mediated m^5^C methylation in HNSCC, but several limitations should be noted. First, the investigation was largely confined to in vitro and in vivo models, which may not fully capture the complexity of HNSCC in human patients. Additionally, the exact mechanisms by which NSUN2 interacts with other epigenetic regulators and signaling pathways are yet to be elucidated. Future study should validate these discoveries in more diverse and larger clinical cohorts and investigate the therapeutic potential of targeting the NSUN2-m^5^C-LAMC2 axis in clinical settings.

## 5. Conclusions

**Figure 8:**
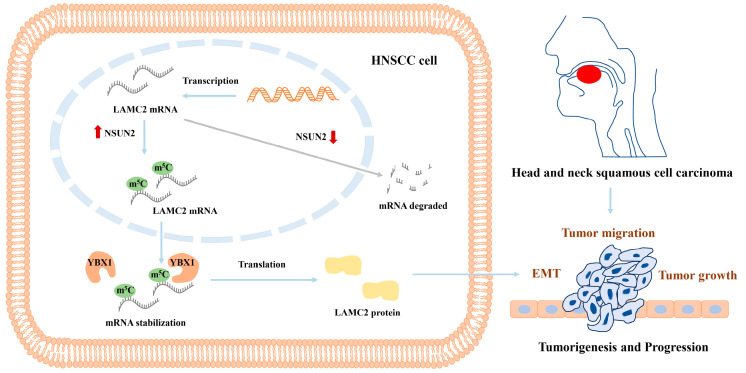
Representative working model. The stability of LAMC2 mRNA is enhanced by NSUN2 and YBX1 through m^5^C modification, subsequently activating the EMT signaling pathway in HNSCC.

In conclusion, the overexpression of LAMC2 in HNSCC is indicative of poor prognosis and promotes tumorigenesis both in vitro and in vivo. NSUN2 and YBX1 enhance the stability of LAMC2 mRNA through m^5^C modification, subsequently activating the EMT signaling pathway in HNSCC (Figure 8). Our findings suggest that the NSUN2-m^5^C-LAMC2 axis represents a promising therapeutic target. Disrupting this regulatory axis could impair the aggressive tumor characteristics of HNSCC, thereby paving the way for novel therapeutic strategies. Future studies should validate our findings in larger and more diverse clinical cohorts and elucidate the mechanisms by which NSUN2 interacts with other epigenetic regulators and signaling pathways. Investigating the therapeutic potential of the NSUN2-m^5^C-LAMC2 axis in clinical settings could provide promising strategies for HNSCC treatment. Additionally, exploring the interaction between m^5^C modification and RNA-binding proteins such as YBX1 may further clarify their combined influence on mRNA stability and tumor progression. Overall, our findings offer significant insights into the molecular mechanisms underlying HNSCC and highlight the NSUN2-m^5^C-LAMC2 axis as a key focus for future research and therapeutic development.

## Figures and Tables

**Table 1:** Demographic data and tumor characteristics of the 131 patients in the SYSU-TFAH cohort.

Variables	N (%) 131	NSUN2-High (n = 65, 49.6%)	NSUN2-Low (n = 66, 50.4%)	*p* Value
Age, year				
Median	57			
Range	29–85			
≤60	80 (61.1)	43	37	0.236
>60	51 (38.9)	22	29	
Gender				
Female	49 (37.4)	26	23	0.542
Male	82 (62.6)	39	43	
Differentiation				
Well	54 (41.2)	23	31	0.206
Moderately	64 (48.9)	33	31	
Poorly	13 (9.9)	9	4	
cT stage				
T1/2	98 (74.8)	46	52	0.290
T3/4	33 (25.2)	19	14	
cN stage				
N0	102 (77.9)	52	50	0.559
N1–3	29 (22.1)	13	16	
cTNM stage				
I/II	83 (63.4)	41	42	0.947
III/IV	48 (36.6)	24	24	

## Data Availability

All datasets associated with this study are available from the corresponding author upon reasonable request. m^5^C-Bis-Seq and mRNA-Seq data are available in the Genome Sequence Archive (GSA) database under accession number HRA007987.

## Supplementary Materials

The following supporting information can be downloaded at: https://www.mdpi.com/article/10.3390/biomedicines12112533/s1, Table S1: The sequences of shRNA, siRNA, and primers.

## Abbreviations

ALYREF, Aly/REF export factor; CDS, coding sequence; DEG, differentially expressed gene; DMS, differential methylation sites; EMT, epithelial–mesenchymal transition; GSA; Genome Sequence Archive; GSEA, Gene Set Enrichment Analysis; H&E, Hematoxylin and eosin; HNSCC, Head and neck squamous cell carcinoma; IHC, Immunohistochemistry; LNM, lymph node metastasis; m5C, 5-Methylcytosine; NSUN2, NOP2/Sun RNA methyltransferase family members 2; qRT-PCR, quantitative real-time PCR; SYSU-TFAH, the First Affiliated Hospital of Sun Yat-sen University; TCGA, The Cancer Genome Atlas; YBX1, Y-box binding protein 1.
