# Supplementary material for: NSUN2 Promotes Head and Neck Squamous Cell Carcinoma Progression by Targeting EMT-Related Gene LAMC2 in an m5C-YBX1-Dependent Manner
Source: Biomedicines. 2024 Nov 6;12(11):2533. doi: 10.3390/biomedicines12112533 (PMC11591655; doi:10.3390/biomedicines12112533)
Supplement: Supplementary file 1 [file biomedicines-12-02533-s001.zip › biomedicines-3207002-supplementary.pdf]

**Supplementary Table S1. The sequences of shRNA, siRNA and primers.**

| **Oligonucleotide** | Sequences |
|---|---|
| NSUN2 shRNA #1 | 5'-CAGTGGAAGGTAATGACGAAA-3' |
| NSUN2 shRNA #2 | 5'-CCCAAGAATGAACGGCTTCAT-3' |
| NSUN2 shRNA #3 | 5'-GAGCGATGCCTTAGGATATTA-3' |
| LAMC2 shRNA #1 | 5'-GCCCTGTCAATGCAACAACAA-3' |
| LAMC2 shRNA #2 | 5'-GCTCACCAAGACTTACACATT-3' |
| LAMC2 shRNA #3 | 5'-CCTGCCAAATTTCTTGGGAAT-3' |
| YBX1 siRNA | 5'-GGAUAUGGUUUCAUCAACATT-3' |
| Human NSUN2 primer | F: 5'-CAAGCTGTTCGAGCACTACTAC-3'<br>R: 5'-CTCCCTGAGAGCGTCCATGA-3' |
| Human LAMC2 primer | F: 5'-GACAAACTGGTAATGGATTCCGC-3'<br>R: 5'-TTCTCTGTGCCGGTAAAAGCC-3' |
| Human GAPDH primer | F: 5'-ACAACTTTGGTATCGTGGAAGG-3'<br>R: 5'-GCCATCACGCCACAGTTTC-3' |
